# Psychological Involvement in the Journey of a Patient with Localized Prostate Cancer—From Diagnosis to Treatment

**DOI:** 10.3390/diseases13100319

**Published:** 2025-10-01

**Authors:** Daniela Mihalcia Ailene, Gabriela Rahnea-Nita, Alexandru Nechifor, Liliana Florina Andronache, Mihaela Emilia Dumitru, Alexandru-Mihai Rebegea, Cristina Stefanescu, Roxana-Andreea Rahnea-Nita, Laura-Florentina Rebegea

**Affiliations:** 1 The Faculty of Medicine and Pharmacy, “Dunarea de Jos” University, 800008 Galati, Romania; daniela.mihalcia@yahoo.com (D.M.A.); alexanfru.nechifor@ugal.ro (A.N.); alexandru.rebegea20@gmail.com (A.-M.R.); laura.rebegea@ugal.ro (L.-F.R.); 2 Specific Disciplines Department, The Faculty of Nursing and Midwifery, “Carol Davila” University of Medicine and Pharmacy, 020021 Bucharest, Romania; gabriela.rahnea-nita@umfcd.ro; 3 M Hospital, 010903 Bucharest, Romania; 4 The Preclinical Department, The Faculty of Medicine, “Carol Davila” University of Medicine and Pharmacy, 020021 Bucharest, Romania; liliana.andronache@umfcd.ro; 5 Radiotherapy Department, “Sfantul Apostol Andrei” Emergency Hospital Galati, 800578 Galati, Romania; mihaeladumitru11@yahoo.com; 6 The Clinical Department, The Faculty of Medicine, “Carol Davila” University of Medicine and Pharmacy, 020021 Bucharest, Romania

**Keywords:** prostate cancer, sexuality, psychological health, emotional support

## Abstract

**Introduction:** Prostate cancer is one of the most common neoplasia in men, and its clinical evolution is highly influenced by psycho-emotional factors, especially in elderly patients. Comorbidities, the perception of one’s identity and its impact on life quality become relevant variables in the therapeutic decision. Sexual dysfunction after treatment along with decreased libido, erectile dysfunction and ejaculatory dysfunction are significant problems in patients with prostate cancer. **Case presentation:** The present study presents the oncological evolution of an elderly patient with a dual diagnosis, prostate adenocarcinoma and lung squamous cell carcinoma, who faced a significant amount of medical and psychological challenges. Reluctance to hormone therapy was closely linked to the fear of sexual dysfunction, a very common reaction in elderly men concerned with maintaining autonomy and intimacy. The peculiarity of the case consists in the interaction between the evolution of the disease, the therapeutic decisions and the psychological impact on the patient. **Discussion:** Androgen deprivation therapy negatively influences multiple aspects of sexual function, significantly impairing the life quality of patients diagnosed with prostate cancer. In this context, therapy through acceptance and commitment is the appropriate one, its main purpose being to change the patient’s relationship with suffering—from struggle and rejection to active acceptance and value of the present. The intervention of the psychologist or the psychotherapist is essential in decision-making counseling, using coping techniques, the clarification of personal values and the involvement of the family in the decision-making process. Oncological psychology helps the patient redefine their life goals and priorities, not just to choose a treatment. **Conclusions:** Sexuality and psychological health are deeply affected by prostate cancer. Psychological flexibility and emotional support can mitigate this negative impact. The integration of therapy through acceptance and commitment in the rehabilitation after treatment increases effectiveness and patient satisfaction.

## 1. Introduction

### 1.1. General Introduction Regarding the Incidence of Prostate Cancer

The second most common type of cancer and the main cause of death due to cancer in men is prostate cancer (PC) [1,2]. Most men are diagnosed with localized prostate cancer (roughly 76%) or with a regional disease, characterized by the extension at the level of the lymph nodes (13%), while approximately 10% present distal metastases upon diagnosis.According to the statistical data collected between 2018 and 2021 (except the year 2020, because of the disturbances caused by the COVID-19 pandemic), it is estimated that approximately 12.9% of men will be diagnosed with prostate cancer throughout their life time [3]. Prostate cancer is a common disorder among elderly men [4], and the increased use of prostate-specific antigen (PSA) testing, along with increased life expectancy, has led to an increased incidence of prostate cancer diagnosis among this population category [5]. Considering that survival rates are high regardless of the treatment of choice, aspects such as treatment-related toxicities that affect the life quality of patients significantly influence their decision-making. Prostate cancer is associated with a lower life quality [6,7]. Sexual dysfunction after treatment, which includes decreased libido, erectile dysfunction and ejaculatory dysfunction, is a significant problem among prostate cancer patients.

### 1.2. The Associated Psychological Impact

In addition to physical effects, there is also a psychological dimension which is often underestimated. According to a systematic study published in January 2025 by the European *Psycho-Oncology* Journal, more than 40% of prostate cancer patients report clinical symptoms such as depression or anxiety within the first 12 months after the diagnosis [8]. These disorders are often related to a fear of relapse, loss of male identity, social isolation and the impairment of sexual function. In addition, patients with a low educational level, advanced age or multiple comorbidities present a significantly higher risk of psychological impairment.

In this context, the integration of scientifically validated psychological interventions becomes essential. Acceptance and Commitment Therapy (ACT) is a method recently promoted in the field of oncological psychology, which was demonstrated in clinical trials between 2024 and 2025 to be effective in reducing psychological stress, in increasing life quality and in supporting patients in adapting to inevitable functional losses [9,10]. ACT does not aim at eliminating emotional symptoms, but at cultivating psychological flexibility, allowing patients to coexist with suffering and uncertainty, while following their personal values [11,12,13].

### 1.3. Diagnosis, Staging and Psychological Impact

Modern imaging methods, such as multiparametric magnetic resonance imaging (mpIRM), ultrasound-guided transrectal biopsy (TRUS-biopsy), digital rectal examination and the serum determination of prostate-specific antigen (PSA) represent main tools in the early diagnosis and staging of prostate cancer. PSA testing, introduced in 1986 as an accessible method to detect asymptomatic prostate cancer, initially led to a significant increase in incidence in developed countries. Subsequently, its use became moderate due to more restrictive guidelines. Although PSA values increase physiologically with age, a level of 4–10 ng/mL is considered borderline and associated with a 25% risk of cancer, while values over 10 ng/mL indicate a risk over 50% [14]. After a positive PSA result, a prostatic biopsy is usually recommended. The biopsy is frequently guided by transrectal ultrasound and/or magnetic resonance imaging (MRI), these also being useful in locating the lesion and estimating the prostate volume. In the event of a positive result, the tumors are subsequently classified according to the Gleason score. The Gleason score is the reference standard scale in the histopathological assessment of prostate adenocarcinoma, based on the tumor glandular architecture. A first-degree rating corresponds to a well-differentiated glandular structure, with minimal cellular atypia, while a fifth-degree rating reflects a completely undifferentiated malignant proliferation, with the complete loss of glandular architecture and cytologic characteristics marked by aggressiveness.

Psychological interventions are essential. Among these, ACT has demonstrated clinical efficiency in supporting cancer patients, including those diagnosed with prostate cancer. ACT promotes acceptance of unavoidable suffering associated with the disease and encourages the active commitment to personal values, thus supporting adaptation to the disease and maintenance of a meaningful life [11]. Studies show that ACT significantly reduces anxiety and depression among cancer patients, increases psychological flexibility and improves the capacity to cope with uncertainty and functional loss [13,15]. Moreover, this form of therapy among men can directly address concerns about masculinity, intimacy and autonomy, supporting the patient in reintegrating personal identity affected by disease [15].

### 1.4. Available Treatments and Androgen Deprivation Therapy’s (ADT) Position in the Therapeutic Plan

The prostate gland is based on androgens for its growth and development. ADT has been a fundamental therapeutic approach since the 1940s [16]. Surgery, radiotherapy and active surveillance are standard treatments for localized prostate cancer. ADT is used in patients with localized recurrent and metastatic PC, with intermediate or high risk, according to the most recent international guidelines [17,18]. In the event of intermediate- to advanced-risk diseases, the therapeutic strategy commonly comprises radiotherapy and ADT—interventions considered essential in multimodal oncologic management [19].

Patients undergoing ADT generally achieve castration levels of circulating testosterone, which is associated with a reduction in tumor charge, improvements of symptoms and increases in survival. However, most cases eventually evolve to a stage of tumor progression under ADT, characterized by an increase in serum values of prostate-specific antigen (PSA), the occurrence of new metastases—most commonly to bone or lymph nodes—or the worsening of clinical symptoms despite maintaining serum testosterone under the castration threshold (<50 ng/dL) [20].

ADT’s mechanism of action is as follows: ADT includes agonists and antagonists of the luteinizing hormone-releasing hormone (LHRH) or bilateral orchiectomy. The hypothalamic–pituitary–gonadal axis is suppressed by the agonists and antagonists of LHRH, which decreases the amount of testosterone in the blood [21,22].

The basic principle of ADT in the management of PC is well established and has remained relatively unchanged since the seminal discovery of Charles B. Huggins, who demonstrated tumor dependence of PC on testosterone nearly eight decades ago [23]. ADT aims to suppress the androgen axis through pharmacological (analogs or antagonists of Gonadotropin release hormone—GnRH) or surgical means (bilateral orchiectomy), with the aim of reducing plasma concentrations of testosterone and dihydrotestosterone (DHT). These androgens are indispensable for the survival and functioning of prostate cells. In an oncological context, malignant prostate cells often exhibit an enhanced activation of the signaling pathway mediated by the androgen receptor, a phenomenon that contributes to tumor proliferation and progression [24].

Androgen receptor pathway inhibitors (ARPIs) have been developed and perfected since the 1960s. The steroidal variants of these agents present a molecular structure similar to endogenous hormones, such as testosterone and progesterone, which can lead to some nonspecific hormonal adverse reactions, which are not directly associated with prostate cancer treatment. Nonsteroidal androgen receptor inhibitors have a different molecular configuration from steroidal compounds, which gives them a superior safety profile regarding progesterone-related side effects. These agents act predominantly as selective androgen receptor antagonists, specifically blocking androgen signaling without inducing unwanted hormonal effects [25].

Advances in understanding the functioning of the androgen receptor (AR), as well as the mechanisms of resistance to androgen deprivation therapy (ADT), have facilitated the occurrence of modern next-generation androgen receptor pathway inhibitors (ARPIs), such as Abiraterone, Enzalutamide, Apalutamide, and Darolutamide [26,27,28].

Although androgen receptor pathway inhibitors (ARPIs) were initially introduced for the treatment of metastatic castration-resistant prostate cancer (mCRPC), their use has significantly expanded as clinical data have accumulated [29,30,31]. Recent studies have revealed that, in combination with androgen deprivation therapy (ADT), these agents also provide benefits in the early stages of the disease. Thus, their efficacy has been demonstrated in non-metastatic castration-resistant prostate cancer (nmCRPC) [32,33], in metastatic forms still sensitive to hormone therapy (mHSPC) [26,28,34,35,36,37,38,39], and, more recently, even in cases of high-risk localized disease, according to the results obtained in the STAMPEDE trial [34].

## 2. Case Presentation

The patient in our study is 75 years old, residing in Galati, Romania, and retired. They presented to the Oncology service with comorbidities, including ischemic heart disease and chronic anemia.

### Pretherapeutic Assessment, Staging and Therapeutic Approach

Pretherapeutic investigation included laboratory tests including complete blood count, inflammatory balance erythrocyte sedimentation rate (ESR or sed rate) and C-reactive protein (CRP), hepatic and renal samples, and determination of initial serum PSA values (14.634 ng/mL upon diagnosis).

MARCH 2024: Prostatic biopsy—the histopathological result confirmed the diagnosis of prostate adenocarcinoma, Gleason score 7 (3 + 4), 2nd-degree group, pattern 4–25%, left perineural invasion.

The pretherapeutic assessment was completed with a CT of the chest and abdomen, MRI of the pelvis and bone scan, being clinically included in stage T2bN0M0, adenocarcinoma Gleason 7, initial PSA 14.63.

The multidisciplinary oncology board proposed ADT plus definitive radiotherapy, which the patient refused for fear of decreased erectile function. The patient expressed reservations about initiating hormonal therapy, expressing fears about its negative impact on sexual function, especially potency. This reluctance is frequently encountered among elderly men who wish to maintain their life quality and personal intimacy even in an oncological context. Psychological counseling was undertaken and it lasted for 6 weeks: one session per week.

The ACT intervention with this patient was structured into six sessions, each targeting one of the core processes of the hexaflex model [11]. The role of these sessions was to help the patient accept the diagnosis and then to get him involved in treatment. In Session 1, psychoeducation and creative hopelessness were introduced to foster acceptance, with a focus on normalizing fears related to sexual dysfunction and shifting from symptom control to value-based living, using metaphors such as the “tug-of-war with cancer” to reframe avoidance. Session 2 emphasized cognitive defusion, addressing catastrophic thoughts around masculinity and intimacy (“I will lose my masculinity”, “I won’t be loved”) through strategies such as labeling thoughts (“I am having the thought that…”) and imagery exercises like “leaves on a stream”, aimed at reducing fusion with distressing cognitions. Session 3 cultivated present-moment awareness through mindfulness practices, including grounding and breath-focused exercises, which were applied both in medical contexts and in marital interactions to decrease avoidance and enhance emotional presence. In Session 4, the focus shifted to self-as-context, distinguishing the patient’s identity from his illness using the chessboard metaphor, thereby reinforcing dignity, autonomy, and perspective-taking. Session 5 involved value clarification, guiding the patient to identify and prioritize core values such as intimacy, companionship, dignity, and autonomy, and translating these into concrete goals, such as maintaining meaningful time with their spouse and continuing hobbies, while reframing concerns about sexual performance within broader intimacy values. Finally, Session 6 focused on committed action, where a personalized plan was developed to support adherence to ADT while sustaining valued life activities, addressing barriers such as fatigue and treatment side effects, and integrating the spouse into a long-term coping strategy, thereby ensuring that therapeutic progress remained grounded in meaningful and sustainable behavior change.

Over the course of the six ACT sessions, the patient’s psychological state was systematically monitored using the Subjective Units of Distress Scale (SUDS), ratings being taken before and after each session. The results showed a progressive and consistent decrease in both anxiety and depressive symptoms after each therapeutic encounter. Initial SUDS values, which were in the high-distress range (7–8/10), gradually decreased session by session, with post-session ratings falling by 2–3 points on average. By the end of the intervention, the patient regularly reported pre-session distress levels around 4–5/10, with post-session levels reduced to 1–2/10. This pattern reflects both immediate session-by-session relief as well as a cumulative long-term improvement in psychological well-being. The patient explicitly reported greater acceptance of their diagnosis, reduced preoccupation with sexual performance, and an increased ability to engage in valued life domains such as maintaining intimacy, gardening, and social interactions.

MAY 2024: CT of the thorax—A pulmonary lump of 1 cm was noticed (right inferior lobe), with recommendation for monitoring

In July, the patient returned for follow-up with PSA = 16.14 ng/mL, and after a discussion with the oncologist and the psychologist, they agreed on ADT treatment, with the decrease in PSA values in October 2024 being down to 0.12 ng/mL.

JULY 2024: Bone scan with 99mTc-HDP

For the assessment of bone dissemination, a whole-body scan was performed in July 2024, with administration of 99mTc-HDP. The examination did not reveal hyper-uptake foci suggestive of bone metastases, but only accumulation at the joint level, corresponding to some degenerative inflammatory modifications. This result offers significant oncologic comfort, confirming that despite the increased PSA values of the patient, the patient did not present bone metastases at the time, which is an important aspect for staging and the choice of subsequent therapeutic conduct.

In the present case, the efficiency of the treatment is reflected in the rapid decrease in PSA values, but monitoring bone density through DEXA assessment remains an important aspect, especially in the presence of cardiovascular comorbidities and advanced age [38,39].

DECEMBER 2024—Progression of pulmonary lesion and undertaking the transthoracic puncture biopsy

After 7 months from the identification of the mass, the follow-up imaging revealed tumor progression. An ultrasound-guided transthoracic puncture biopsy was recommended for the histopathological analysis. The patient postponed the transthoracic biopsy for 2 months, until February 2025.

FEBRUARY 2025—The pathological result and the recommendation for immunohistochemistry

The analysis report revealed well-differentiated bronchopulmonary squamous cell carcinoma (G1), the diagnosis being also supported by an immunohistochemical examination (molecular markers: negative EGFR, negative ALK, PDL 1).

MARCH 2025—Right inferior lobectomy with mediastinal lymphadenectomy was performed, with a histopathological result of well-differentiated keratinized squamous cell carcinoma (G1).

Classification pTNM: pT4N0

Classification TNM pT4N0 revealed an extensive primary tumor (8.6/7.2/5 cm), without lymph node involvement, with severe local invasive potential and reserved prognosis [40].

It is worth mentioning that a postoperative evaluation was performed, including a brain CT scan, which ruled out the presence of secondary lesions.

We are discussing the case of a patient with two synchronous neoplastic sites that require a multidisciplinary approach for each neoplastic site.

The multidisciplinary oncology board decided to continue the androgen deprivation therapy (ADT) and to initiate adjuvant chemotherapy, given the biological profile of the patient and their comorbidities.

**Figure 1:**
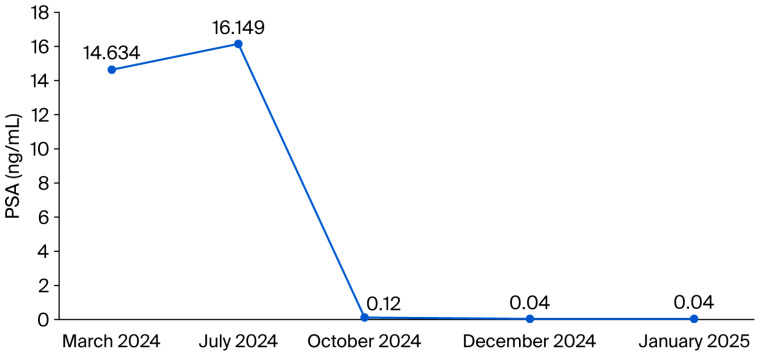
PSA evolution in time.

In July 2024, given the increased PSA value to 16.149 ng/mL, the patient accepted the beginning of the androgen deprivation therapy. The therapeutic response was prompt and marked: upon the October 2024 assessment, the PSA value had decreased to 0.12 ng/mL, and the recorded testosterone value was at castration level, confirming the effectiveness of the hormonal therapy. In the following months, PSA values became almost undetectable, remaining below 0.05 ng/mL until January 2025, which suggests clear biochemical remission (Figure 1).

Up to the present moment (August 2025), the patient has undergone three rounds of adjuvant chemotherapy (Gemcitabine plus Carboplatin), with Pegfilgrastim for the prophylaxis of febrile neutropenia, without hematologic or non-hematologic toxicities for lung cancer. At the same time, the patient continues androgen deprivation therapy.

## 3. Discussion

### 3.1. Sexual and Systemic Side Effects of ADT

The treatment of prostate cancer is designed to relieve the symptoms of the disease and improve the patient’s quality of life [41,42,43]. In the context of hormonal treatment, it should be mentioned that ADT is commonly associated with systemic adverse effects, including reduced mineral bone density, sarcopenia, dyslipidemia, fatigue, and marked sexual dysfunction.

ADT negatively impacts many aspects of sexual function, potentially significantly compromising the life quality of patients diagnosed with prostate cancer (PC). ADT may cause penis length shortening and testicular atrophy, and in association with body mass changes, it decreases muscle mass and facilitates the occurrence of gynecomastia. It can negatively affect body image perception, thus contributing to sexual dysfunction and less intimacy between couples [44,45]. The link between ADT administration, decreased libido and erectile dysfunction is well documented. A recent meta-analysis revealed that the use of ADT increases the risk of decreased libido by 5–6 times and the risk of erectile dysfunction by approximately 3 times [45].

ADT is a fundamental component in the management of prostate cancer; it is associated with a wide range of side effects. According to a study conducted by Donovan KA et al., which included 231 patients who underwent either androgen deprivation therapy or prostatectomy, it was revealed that the most prevalent documented side effect was erectile dysfunction (ED), reported in approximately 70–90% of the cases. The results of this study also highlight other common manifestations, including decreased libido, anorgasmia or difficulty achieving orgasm, and penis length shortening and testicular atrophy, all of these contributing to the significant deterioration of sexual and psychological life quality of the cancer patient [19].

A recent study conducted by Wibowo et al. revealed that 82% of patients undergoing ADT reported erectile dysfunction, and 64% mentioned decreased libido [46] (Table 1).

Given the fact that most patients are sexually active at the time of diagnosis of prostate cancer, the side effects associated with ADT may be a significant burden for those who wish to maintain an active sex life during and after the treatment. It is essential to highlight that, besides the direct impact of ADT, erectile function is also influenced by a series of comorbid factors commonly seen in elderly men, including advanced age, such as diabetes mellitus, arterial hypertension, cardiovascular diseases, dyslipidemia, tobacco and alcohol consumption, certain drug treatments and obesity [47].

### 3.2. The Psycho-Sexual Impact and the Importance of Intervention

A decreased libido may lead to diminished physical and emotional intimacy, generating relational distress and negatively affecting life quality. Therefore, it is recommended to implement some psycho-sexual interventions based on adjusting post-therapeutic expectation and reducing the impact of ADT’s adverse effects, with the active involvement of both partners in the adaptation process [19,48].

The World Health Organization (WHO) defines sexuality as “a central aspect of the human being throughout life and encompasses sex, gender identities and roles, sexual orientation, erotism, pleasure, intimacy and reproduction” [49]. This definition highlights the multidimensional and essential nature of sexuality in the life of each individual. In situations in which the individual is faced with a serious health problem, such as the diagnosis of cancer, their whole sexuality may be disrupted, thus significantly affecting life quality and psychological balance.

In the case of prostate cancer, these disruptions take on a specific value, since the affected organ has a main role in male sexuality. Many patients develop fears related to the loss of potency, which may lead to emotional withdrawal, feelings of inadequacy and deterioration of intimate relationships. Studies reveal that the partners of patients with prostate cancer often report a significant decrease in sexual satisfaction, this being influenced both by the sexual dysfunction of their partner and by the natural factors of aging and side effects of the oncological treatment [50].

These concerns are part of a broader spectrum of psychological suffering, since the psychological impact of prostate cancer is profound. Patients are exposed to a high risk of developing major affective disorders, such as depression and anxiety, and may exhibit suicidal behaviors. One of the most relevant studies in the field conducted a systematic review and meta-analysis of the existing literature in order to estimate the prevalence of such disturbances among prostate cancer patients [51]. The study only included observational research that used validated methods to assess depression, anxiety and suicidal after the diagnosis, analyzing data collected from databases such as MEDLINE, Scopus, PsycInfo and Cochrane Library.

The results of this thorough analysis revealed a prevalence of 5.81% for clinical depressive disorders (based on 11 studies with a total number of 655,149 patients). Moreover, 17.07% of these patients exhibited significant depressive symptoms, although below the clinical diagnostic threshold (based on 76 studies including 32,339 patients). Anxiety-related symptoms were seen in 16.86% of the patients (56 studies, 24,526 patients), and 9.85% reported recent suicidal thoughts (8 studies, 6173 patients). Furthermore, ADT, commonly used in the treatment of prostate cancer, was associated with a significant increase in depression (OR = 1.42) and depressive symptoms (OR = 3.14) [51].

Given these challenges, certain psychological variables, such as psychological flexibility and self-esteem, seem to mediate the negative side effects of oncological stress on intimate relationships. These internal resources contribute to maintaining a satisfactory relationship, even in conditions of suffering and medical uncertainty [52]. For this reason, psychological interventions aimed at improving psychological flexibility may have a significant impact on the relational functioning of couples affected by prostate cancer [53].

A common consequence of the surgical treatment of prostate cancer, especially of radical prostatectomy, is ED, which negatively affects male identity, self-esteem and sexual life. Although penis rehabilitation therapy is a recommended intervention for the recovery of erectile function, many patients do not adhere to this, especially if it consists of penial injections, an effective but invasive method that is often perceived as embarrassing [54,55].

In this context, a study explored the efficacy of Acceptance and Commitment Therapy for Erectile Dysfunction (ACT-ED) in facilitating the consistent use of penile injections after radical prostatectomy [52]. A total of 53 men were randomly assigned to two groups: one group benefited from ACT-ED (n = 26), consisting of four therapeutic sessions and three support telephone calls, while the other group (Enhanced Monitoring—EM; n = 27) benefited from standard care and seven monitoring telephone calls made by a sexual medicine specialist.

The results obtained after 4 months indicated superior adherence in the ACT-ED group, with a mean value of 1.7 injections/week, compared to 0.9 in the EM group. Moreover, 44% of the patients in the ACT-ED group followed the recommended regimen (minimum 2 injections per week) compared to only 10% in the EM group [19] After 8 months, the use of injections was still more frequent in the ACT-ED group (1.2 vs. 0.7 injections/week). The patients in this group also reported greater satisfaction with their erectile dysfunction treatment, improved sexual self-esteem, less ED-related discomfort, and less regret about their choice of oncologic treatment.

These data indicate that integrating ACT techniques into medical practice may provide significant benefits for the patients experiencing ED after prostatectomy, contributing to the reduction in psychological barriers and to the increase in sexual rehabilitation treatments [52,54,55].

### 3.3. The Psychological Impact and the Benefits of Acceptance and Commitment Therapy (ACT) in the Case of Our Study Patient

The patient in our study, aged 75, faced a significant amount of medical and psychological challenges: two oncological diagnoses (prostatic adenocarcinoma and lung squamous cell carcinoma), chronic comorbidities and treatments with potentially severe impacts on life quality.

The patient’s psychosocial background provides essential context for understanding the psycho-oncological dimensions of their case and highlights the nuanced interplay between resilience and vulnerability in their adjustment to illness. Recently remarried, the patient simultaneously carries the unresolved grief of having lost their first wife to cancer, a duality that renders intimacy and relational security both highly valued and emotionally complex. This biographical reality situates the patient’s concerns about treatment-induced sexual dysfunction within a broader framework of identity, loss, and the desire to preserve closeness in their new relationship. At the same time, their psychosocial resources, including strong social ties with friends and active engagement in meaningful hobbies such as gardening and playing chess, function as resilience scaffolds, buffering against isolation and rumination and offering pathways for sustaining quality of life beyond illness. Yet this ambivalence, valuing intimacy while fearing the impact of therapy on sexual function, illustrates the central psychological challenge of reconciling bodily losses with personal values. In this context, an intervention such as ACT becomes particularly relevant, as it allows the patient to reframe distressing cognitions, accept the inevitability of certain functional impairments, and commit to maintaining intimacy and dignity in ways that transcend sexual performance. Addressing this psychosocial complexity is therefore not only critical for mitigating psychological distress but also for strengthening adherence to oncological treatment and fostering long-term adaptation.

In this context, the psychological dimension plays an important role in treatment adherence, acceptance of disease and maintenance of emotional balance.

Psychological counseling for men with prostate cancer in an ACT (Acceptance and Commitment Therapy) framework focuses on addressing the distress caused by sexual dysfunction, threats to masculinity, intimacy concerns, and treatment-related anxiety by cultivating psychological flexibility through six interrelated processes. Acceptance helps patients reduce avoidance of grief, shame, and loss by learning to sit with painful emotions rather than fight them, while cognitive defusion teaches them to see thoughts such as “I am no longer a man” as mental events rather than facts. Present-moment awareness practices, including mindfulness and grounding, reduce rumination and help patients stay connected during medical procedures or intimate moments, and self-as-context techniques such as the chessboard metaphor enable them to perceive themselves as more than their illness or bodily changes. Value clarification guides patients in identifying what truly matters—often emotional closeness, dignity, and companionship—so that intimacy can be redefined beyond sexual performance, and committed action supports small, realistic steps aligned with those values, such as nurturing affectionate touch with a partner or adhering to treatment for the sake of loved ones. Emerging evidence from ACT-ED trials, partner-based interventions, and digital ACT programs shows improvements in treatment adherence, intimacy satisfaction, and reduced distress, suggesting that ACT provides a powerful therapeutic framework for helping men with prostate cancer adapt to functional losses while maintaining identity, meaning, and life quality.

The specialty literature highlights the fact that elderly cancer patients are predisposed to depressive symptoms, anxiety, adaptation disorders and a low perception of life quality [53]. Particularly, in the case of men diagnosed with prostate cancer, the impact on self-esteem and the fears related to loss of potency may increase emotional distress and reduce therapeutic collaboration [52] In the case of the patient in our study, reluctance to undergo hormonal therapy was closely related to the fear of sexual dysfunction, a common reaction among elderly men concerned with maintaining autonomy and intimacy [53].

Acceptance and Commitment Therapy (ACT) is a modern psychological intervention, scientifically documented, which aims at developing psychological flexibility by accepting difficult internal experiences and engaging in behaviors guided by personal values [11]. ACT proved efficiency in reducing anxiety, depression and psychological distress among cancer patients, also contributing to increasing life quality and treatment engagement [11,53].

This approach is particularly appropriate in a geriatric context, where the main goal is not avoiding psychological pain, but changing the patient’s relationship with suffering—from struggle and rejection into active acceptance and value of the present [11]. In the case of our patient, ACT may facilitate the acceptance of the diagnosis and treatment and clarify personal values (such as dignity, significant relationships and autonomy) but also reduce anxiety linked to disease-related losses [11,53].

Recent evidence has further highlighted the profound psychological burden of prostate cancer [55]. A 2025 systematic review and meta-analysis of over 60 observational studies revealed that men with prostate cancer present a significantly increased risk of both suicide mortality (pooled SMR = 1.25, 95% CI 1.17–1.34) and suicidal ideas/attempts (pooled RR/HR = 1.70, 95% CI 1.29–2.24) compared to controls. The risk was especially pronounced in patients with advanced disease or those experiencing severe functional losses, underscoring how the interplay of treatment side effects, loss of sexual function, and identity disruption may lead to existential despair. These findings are crucial for psycho-oncological practice, as they demonstrate that beyond managing depression and anxiety, clinicians must actively screen for suicide in men with prostate cancer, particularly those undergoing androgen deprivation therapy or facing relational strain.

In the context of our case, these insights suggest that ACT [11] interventions may serve not only to improve life quality, but also as a potential protective factor against suicide. By fostering psychological flexibility, ACT helps patients reframe self-critical thoughts (“I am no longer a man”) as transient mental events, while grounding them in values such as dignity, companionship, and legacy. This reframing may reduce the intensity of suicidal ideas by reconnecting patients with meaning despite losses. Moreover, ACT’s emphasis on committed action—such as maintaining intimacy in non-sexual ways or engaging with meaningful hobbies and social roles—aligns with empirical findings that value-driven behavior buffers against hopelessness and suicide risk.

Thus, integrating ACT into oncological care has implications beyond symptom relief: it addresses an urgent public health concern by mitigating the elevated risk of suicide in prostate cancer patients. This case highlights how systematic suicide risk monitoring, combined with value-oriented psychotherapy, can create a more comprehensive psycho-oncological approach, ensuring that treatment supports not only survival but also the preservation of purpose and psychological safety [54].

Thus, in cases with such a high medical and emotional burden, ACT provides an effective and clinically validated therapeutic framework, with the potential to support the patient in the adaptation process and to preserve their life quality, even in cases of poor oncological prognosis [11,53,55].

## 4. Conclusions

This case reflects the complexity of oncological management in elderly patients with multiple pathologies, synchronous neoplasms and associated risk factors. Although the patient was initially reluctant, they benefited from hormonal therapy and thus achieved an excellent biochemical response. The lack of bone metastases confirmed upon bone scan adds a favorable element to the oncological prognosis, and effective tumor control permits the planning of subsequent therapeutic interventions, targeted and adapted to the biological and the functional profile of the patient.

It also highlights the major impact of dual oncological diagnosis on therapeutic planning, life quality and emotional balance of the elderly patient. The choice of treatment was deeply influenced not only by the biological state of the patient, but also by their perception of the disease, the fear of adverse reactions and insufficient emotional support from their family. In this context, early psychological intervention proved to be essential. The ACT-based program, monitored with Subjective Units of Distress Scale (SUDS) before and after each session, demonstrated a consistent reduction in anxiety and depressive symptoms, with immediate improvements after every session and cumulative long-term benefits. This translated into greater acceptance of the therapy, better treatment adherence, and enhanced engagement with valued life domains. Therefore, the case reinforces the importance of integrating psychological and social care into oncological management, especially when treatment decisions are fragile and prognosis is poor. A patient-centered approach, with close interdisciplinary collaboration, not only supports medical decision-making but also secures emotional balance, life quality, and long-term therapeutic compliance. This case reinforces the importance of integrating psychological and social care into oncological management, especially in situations in which therapeutic decisions are fragile and the prognosis is poor. Therefore, the current study highlights the need for a patient-centered approach, in which the medical teams collaborate in an interdisciplinary manner, not only regarding treatment planning, but also in terms of decision-making, in the provision of emotional and educational support to the patient and their family.

## Figures and Tables

**Table 1:** The most common side effects of androgen deprivation therapy (ADT).

Sexual Side Effects	Other Systemic Side Effects
Erectile dysfunction (ED)	Hot flushes
Low libido	Loss of bone mineral density/loss of muscle mass/sarcopenia
Penis shrinkage	Cardiovascular effects/dyslipidemia
Testicular atrophy	Psychiatric disorders/cognitive disorders
Hypogonadism	Weight gain/gynecomastia
Delayed orgasm/anorgasmia	Decreased energy/tiredness

## Data Availability

The original contributions presented in this study are included in the article. Further inquiries can be directed to the corresponding authors.

## Abbreviations

The following abbreviations are used in this manuscript:

PC	prostate cancer
PSA	prostate-specific antigen
ACT	Acceptance and Commitment Therapy
ACT-ED	Acceptance and Commitment Therapy for Erectile Dysfunction
mpIRM	multiparametric magnetic resonance imaging
MRI	magnetic resonance imaging
ADT	androgen deprivation therapy
ARPIs	androgen receptor pathway inhibitors
TRUS-biopsy	ultrasound-guided transrectal biopsy
GnRH	Gonadotropin release hormone
LHRH	luteinizing hormone-releasing hormone
ESR	erythrocyte sedimentation rate
CRP	C-reactive protein
ED	erectile dysfunction
WHO	World Health Organization
EM	Enhanced Monitoring
